# Synergy Between Vitamin D and Sex Hormones in Respiratory Functionality of Patients Affected by COVID-19

**DOI:** 10.3389/fphar.2021.683529

**Published:** 2021-05-13

**Authors:** Daniela Peruzzu, Maria Teresa Pagano, Marina Pierdominici, Anna Ruggieri, Andrea Antinori, Gianpiero D’Offizi, Nicola Petrosillo, Fabrizio Palmieri, Pierluca Piselli, Evangelo Boumis, Stefania Notari, Emanuele Nicastri, Chiara Agrati, Giuseppe Ippolito, Maria Cristina Gagliardi, Maria Rosaria Capobianchi, Elena Ortona, INMI-ISS COVID-19 Team

**Affiliations:** ^1^ Center for Gender Specific Medicine, Istituto Superiore di Sanità, Rome, Italy; ^2^ National Institute for Infectious Diseases, Lazzaro Spallanzani, IRCCS, Rome, Italy

**Keywords:** SARS-CoV-2, vitamin D, sex hormones, gender, sex differences

## Abstract

The outcome of COVID-19 appears to be influenced by vitamin D status of population. Although epidemiological data indicate that COVID-19 produces more severe symptoms and higher mortality in elderly in comparison to young patients and in men in comparison to women to date sex and age differences in vitamin D status in infected patients have not been evaluated yet. In this study we evaluated the levels of circulating 25(OH)D in patients hospitalized for COVID-19 divided accordingly to their sex and age. We also correlated 25(OH)D levels with patient’s respiratory status (i.e., PaO2/FiO2 ratio) and with sex hormones plasma levels to analyze the potential relationship of these parameters. We found no significant differences in plasma levels of 25(OH)D between pre- and post-menopausal female patients and age matched male patients. Interestingly, the 25(OH)D plasma levels positively correlated to PaO2/FiO2 ratio only in young patients, regardless of their sex. We also found a significantly positive correlation between 17β-estradiol and 25(OH)D in elderly women and between testosterone and 25(OH)D in elderly men, supporting the role of sex hormones in maintaining 25(OH)D levels. In conclusion, we suggest that a synergy between vitamin D and sex hormones could contribute to the age-related outcome of COVID-19.

## Introduction

The outcome of coronavirus disease 2019 (COVID-19) appears to be influenced by the interaction among genetic, hormonal and environmental factors. In this context, low levels of circulating 25-hydroxyvitamin D [25(OH)D], the biomarker of vitamin D status, represent a risk factor for COVID-19 and 1,25(OH)2 vitamin D(3) (the active metabolite of vitamin D) seems to play a protective role in this disease by controlling the cytokine storm, enhancing the production of antimicrobial peptides and maintaining the integrity of the epithelium (Pagano et al., 2020; Hutchings et al., 2021). Moreover, 1,25(OH)2 vitamin D(3), by increasing the expression of angiotensin-converting enzyme (ACE)2, the functional receptor for SARS-CoV-2, induces ACE2/Ang‐(1-7)/MasR axis activity and inhibits renin and the ACE/Ang II/AT1R axis. This process leads to the protection against acute lung injury/acute respiratory distress syndrome (Xu et al., 2017; Gagliardi et al., 2020; Kumar et al., 2020; Malek Mahdavi, 2020). In the same vein, 17β-estradiol increases the expression of ACE2 also playing a protective role in COVID-19 (Seeland et al., 2020). Although epidemiological data indicate that COVID-19 produces more severe symptoms and higher mortality in elderly in comparison to young patients and in men in comparison to women (Pagano et al., 2020; Chen et al., 2021), to date, sex, and age differences in vitamin D status in infected patients have not been evaluated yet. Hence aim of this study was to analyze the levels of circulating 25(OH)D in patients hospitalized for COVID-19 divided accordingly to their sex and age. We also correlated 25(OH)D levels with patient's respiratory status and with sex hormones plasma levels to analyze the potential relationship of these parameters.

## Methods

To address this issue, we measured plasma levels of 25(OH)D, testosterone and 17β-estradiol from 160 adult patients (80 females and 80 males divided in four subgroups: A, 40 pre-menopausal females under 45 years; B, 40 males under 45 years; C, 40 post-menopausal females over 55 years; D, 40 males over 55 years) admitted to Lazzaro Spallanzani Hospital, Rome, Italy between March and September 2020, positive for SARS-CoV-2 by RT–PCR from nasopharyngeal swabs, and able to provide informed consent. Plasma samples were collected at clinical admission. Partial pressure of oxygen/inspired oxygen concentration ratio (PaO2/FiO2 ratio), representing a valuable clinical measure of the patient’s respiratory status, were recorded during clinical admission. ELISA kits for 25(OH)D (MyBioSource, San Diego, CA, United States) testosterone (R&D Systems, Minneapolis, MN, United States) and 17β-estradiol (Abcam, Cambridge, United Kingdom) were used according to manufactures’ instructions. Statistical analysis was performed by the Mann–Whitney *U* test and Spearman’s rank correlation using GraphPad Prism (GraphPad Software, San Diego, CA, United States). A *p* value <0.05 was considered statistically significant.

## Results

**FIGURE 1:**
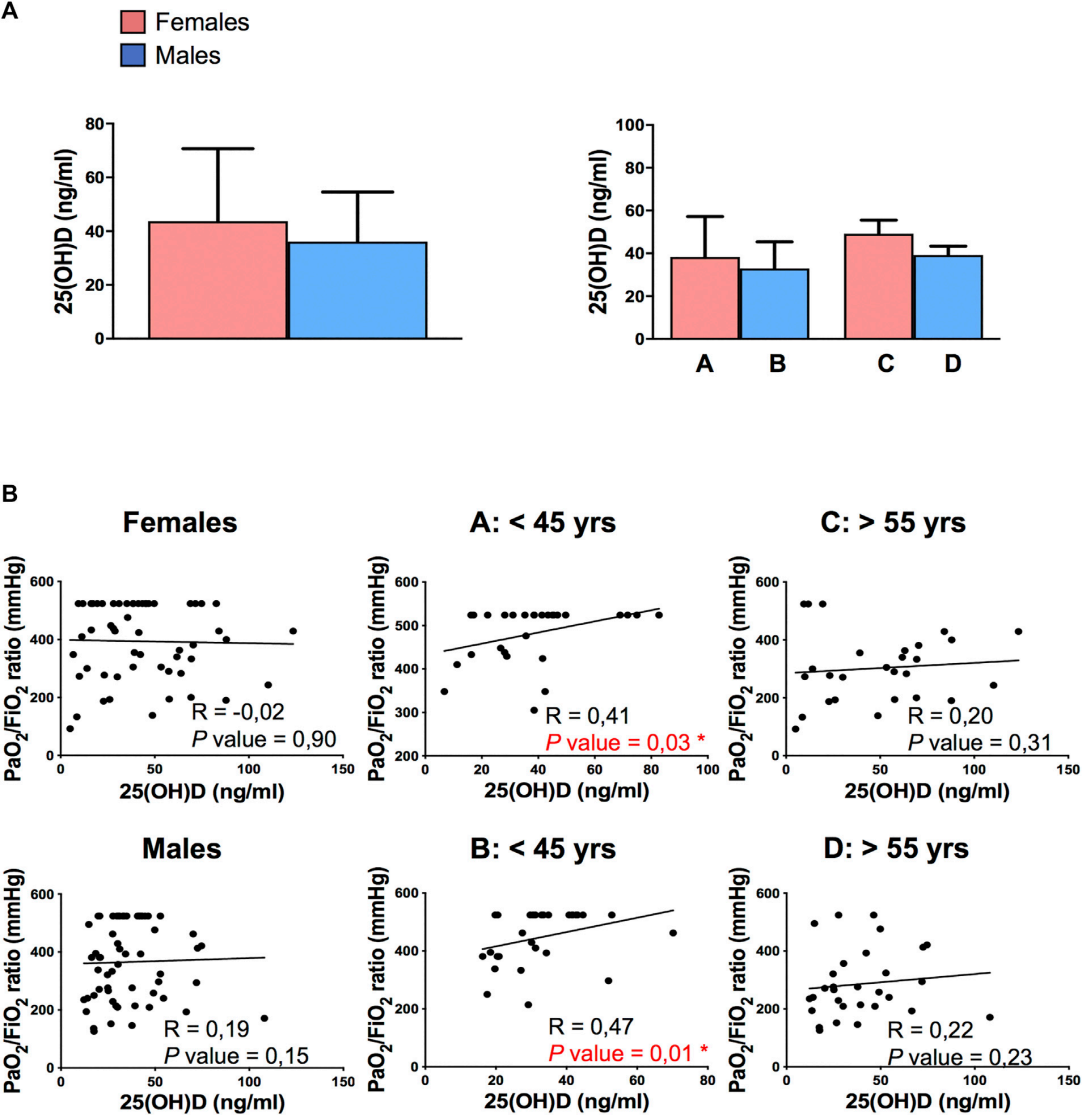
Detection of plasma levels of 25(OH)D and its correlation with PaO2/FiO2 ratio in COVID‐19 patients. **(A)** Analysis of plasma levels of 25(OH)D detected into female and male patients (left panel) and among the 4 subgroups of patients: A, 40 pre-menopausal females under 45 years; B, 40 males under 45 years; C, 40 post-menopausal females over 55 years; D, 40 males over 55 years. Data referred to plasma levels of 25(OH)D are reported as mean ± SD. **(B)** Correlation and linear regression analysis of plasma levels of 25(OH)D and PaO2/FiO2 ratio in female and male patients categorized into four groups: A, 40 pre-menopausal females under 45 years (A: < 45 yrs); B, 40 males under 45 years (B: < 45 yrs); C, 40 post-menopausal females over 55 years (C: > 55 yrs); D, 40 males over 55 years (D: > 55 yrs). A significantly positive correlation between plasma levels of 25(OH)D and PaO2/FiO2 ratio is observed in subgroups A and B. The Spearman's rho (R) and *P* values were determined using the Spearman's rank correlation analysis. Solid lines represent best fits as estimated by linear regression analysis. *, *P* < 0.05 was considered statistically significant.

**FIGURE 2:**
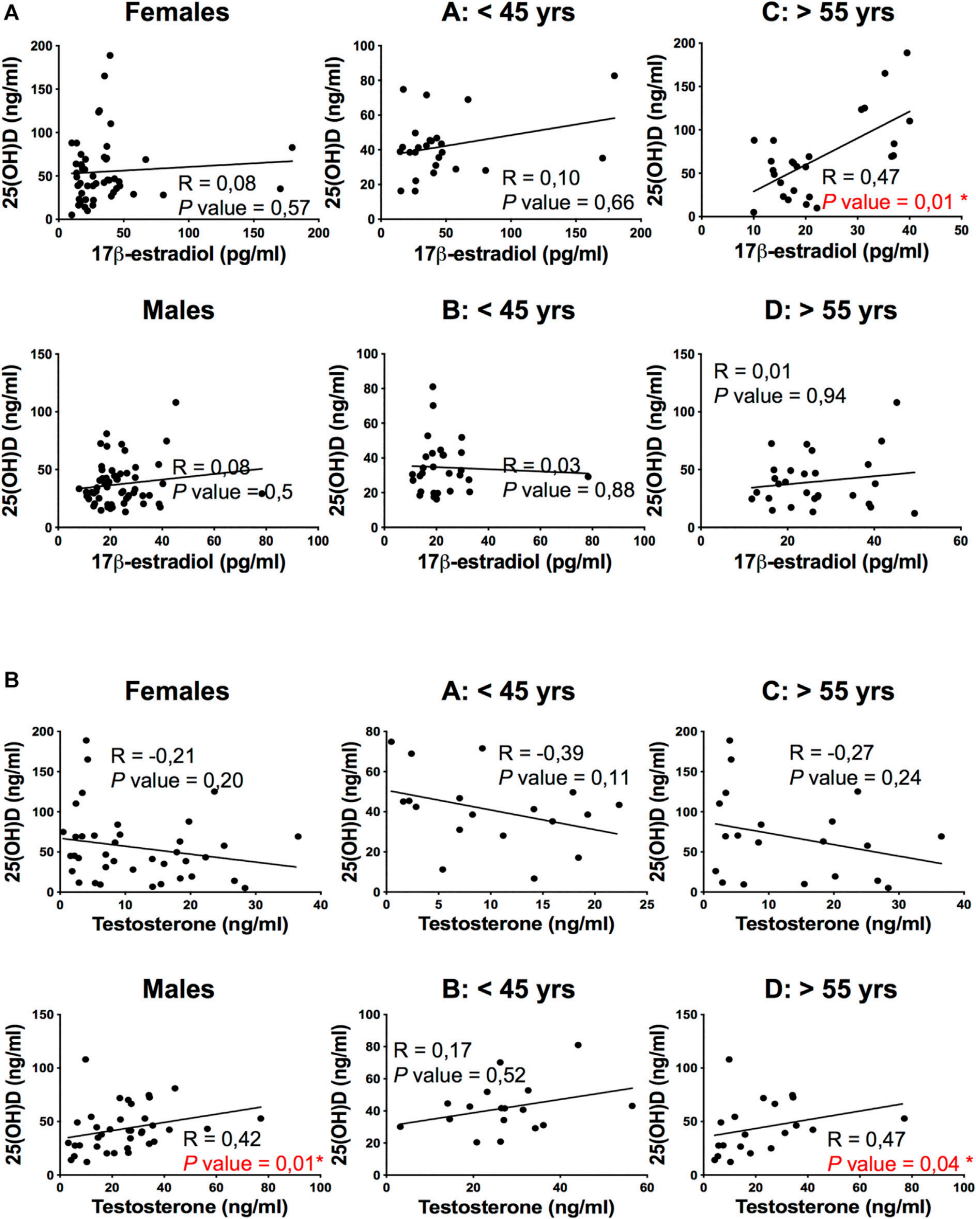
Correlation of plasma 25(OH) vitamin D levels with 17β-estradiol and Testosterone in COVID-19 patients. **(A)** Correlation and linear regression analysis of plasma levels of 25(OH)D and 17β-estradiol in female and male patients categorized into four groups: A, 40 pre-menopausal females under 45 years (A: < 45 yrs); B, 40 males under 45 years (B: < 45 yrs); C, 40 post-menopausal females over 55 years (C: > 55 yrs); D, 40 males over 55 years (D: > 55 yrs). A significantly positive correlation between plasma levels of 25(OH)D and 17β-estradiol is observed in subgroup C. **(B)** Correlation and linear regression analysis of plasma levels of 25(OH)D and testosterone into four groups. A significantly positive correlation between plasma levels of 25(OH)D and testosterone levels is observed in males and in subgroup D. The Spearman's rho (R) and *P* values were determined using the Spearman's rank correlation analysis. Solid lines represent best fits as estimated by linear by linear regression analysis. *, *P *< 0.05 was considered statistically significant.

No significant differences in plasma levels of 25(OH)D were detected between female and male patients and among the four subgroups of patients divided according to sex and age (Figure 1A). Interestingly, the 25(OH)D plasma levels positively correlated to PaO2/FiO2 ratio only in young patients (subgroups A and B, *p* = 0,03 and *p* = 0,01 respectively, Figure 1B), regardless of the sex of patients. The here observed age-dependent vitamin D association with respiratory functionality in COVID-19 patients suggested the involvement of sex hormones in vitamin D effects. Hence, we correlated 25(OH)D levels with sex hormones plasma levels and we observed a significantly positive correlation between 17β-estradiol and 25(OH)D in elderly women (subgroup C, *p* = 0.01, Figure 2A) and between testosterone and 25(OH)D in elderly men (subgroup D, *p* = 0.04, Figure 2B), supporting the role of sex hormones in maintaining 25(OH)D levels.

## Discussion

In this study we analyzed the 25(OH)D plasma levels in patients with COVID-19 divided accordingly to their sex and age and we found a positive correlation between vitamin D status and respiratory functionality only in young patients, regardless of their sex. This result was consistent to previous studies on the association of serum 25(OH)D concentration with the improved lung function, in both females and males (Ganji et al., 2020). We asked whether in young patients, sex hormones could impact vitamin D status and effects. Actually, estrogen has been suggested to enhance vitamin D actions, by increasing the expression of the nuclear vitamin D receptor gene (Cheema et al., 1989) and by decreasing the expression of CYP24A1, the cytochrome P450 component of the 25-hydroxyvitamin D(3)-24-hydroxylase enzyme which catabolizes the active form of vitamin D. In turn, vitamin D influences peripheral estrogen metabolism modulating in a tissue-specific way the function of the cytochrome P450 19 aromatase (CYP19), the enzyme that produces 17β-estradiol from testosterone (Lundqvist et al., 2011). Moreover, low levels of vitamin D have been also independently associated with low levels of testosterone in healthy middle-aged men (Giagulli et al., 2021; Papadopoulos et al., 2021). Accordingly, we found a significantly positive correlation between 17β-estradiol and 25(OH)D in elderly women and between testosterone and 25(OH)D in elderly men supporting the role of sex hormones in preserving 25(OH)D levels. A relationship between hormones of the hypothalamic–pituitary–testicular (HPT) axis and vitamin D status has been established (Lee et al., 2012). In this context, further studies are needed to evaluate gonadotropin levels in male patients in order to clarify the role of vitamin D/HPT axis in COVID-19.

In conclusion, it is tempting to hypothesize that the synergy between vitamin D and sex hormones could contribute to the age-related outcome of COVID-19. Controlled studies are needed to determine the role of vitamin D supplementation in combination with estrogens or androgens in improving respiratory function of female and male patients, respectively.

All in all, the collection, analysis and report of data on COVID-19 disaggregated by sex and age seem to be a duty to achieve the appropriateness of the prevention, treatment and control of the disease.

## INMI-ISS COVID-19 Team

Simona Anticoli, Maria Bellenghi, Veronica Bordoni, Marta Camici1, Rita Casetti, Carlotta Cerva, Pierangelo Chinello, Eleonora Cimini, Davide Roberto Donno, Maria Luisa Dupuis, Francesca Faraglia, Katia Fecchi, Roberta Gagliardini, Germana Grassi, Rachele Di Lorenzo, Manuela Macchione, Gaetano Maffongelli, Gianfranco Mattia, Eugenia Milozzi, Silvia Mosti, Giada Pontecorvi, Rossella Puglisi, Alessandra Sacchi, Eleonora Tartaglia, Serena Vita.

## Data Availability

The data that support the findings of this study are available from the corresponding author.
